# A Novel N‐Terminal *PRPF6* Variant in Autosomal Dominant Retinitis Pigmentosa

**DOI:** 10.1002/ccr3.71783

**Published:** 2026-01-22

**Authors:** Na Li, Yalong Dang

**Affiliations:** ^1^ Department of Ophthalmology Sanmenxia Eye Hospital/Sanmenxia Central Hospital Affiliated to Henan University of Science and Technology Sanmenxia China; ^2^ Henan International Joint Laboratory of Outflow Engineering, Sanmenxia, Central Hospital, School of Medicine Henan University of Science and Technology Sanmenxia China

**Keywords:** autosomal dominant inheritance, N‐terminal variant, PRPF6, retinitis pigmentosa, spliceosome

## Abstract

This report identifies the first N‐terminal *PRPF6* variant (c.514C>T) as a cause of autosomal dominant Retinitis Pigmentosa. This novel variant is associated with progressive peripheral vision loss but notably preserved central visual acuity, suggesting a distinct phenotypic expression compared to C‐terminal variants.

## 1 Introduction

Retinitis pigmentosa (RP) is a hereditary blinding eye disease characterized by the progressive apoptosis of photoreceptor cells [1]. More than 100 genes are implicated in RP, with approximately 5%–10% of cases following an autosomal dominant (adRP) inheritance pattern, often involving genes encoding splicing regulatory factors [2]. The *PRPF6* (OMIM: 613979) gene, a core component of the U5 small nuclear ribonucleoprotein (snRNP) complex, plays a key role in pre‐mRNA splicing [3]. Pathogenic variants in this gene disrupt splicing, leading to retinal degeneration.

Previously reported pathogenic variants in *PRPF6* are exclusively located in the C‐terminal functional domain [4]. No pathogenic variants have been reported in the N‐terminal region, which accounts for 40% of the protein's full length.

This case reports a 41‐year‐old female RP patient carrying a novel N‐terminal *PRPF6* variant (c.514C>T), expanding the genetic spectrum of PRPF6 and offering new perspectives on the genotype–phenotype correlations in adRP. The study adhered to the Declaration of Helsinki and was approved by the Ethics Committee of Sanmenxia Central Hospital (20250123). Written informed consent from all participants was obtained according to the journal guidelines.

## 2 Case Presentation

A 41‐year‐old female presented with a chief complaint of blurred vision and significant night vision difficulties persisting for 30 years.

### 2.1 History of Present Illness

The patient reported insidious‐onset nyctalopia and subjective visual decline starting at age 11. Despite preserved high‐contrast central vision, she experienced difficulties likely attributable to peripheral field loss. Over the past 4 years, her symptoms worsened, prompting a comprehensive evaluation. Following informed consent, a familial investigation was conducted. Ophthalmic examinations confirmed RP findings in the proband's mother, maternal aunt, younger sister, and daughter, all of whom reported similar symptoms. Unaffected family members exhibited normal ocular findings.

### 2.2 Ophthalmic Examination

The patient's best corrected visual acuity (BCVA) was 20/20 (Snellen) in both eyes. The anterior segment examination, including cornea, lens, and anterior chamber depth, was unremarkable bilaterally.

**FIGURE 1:**
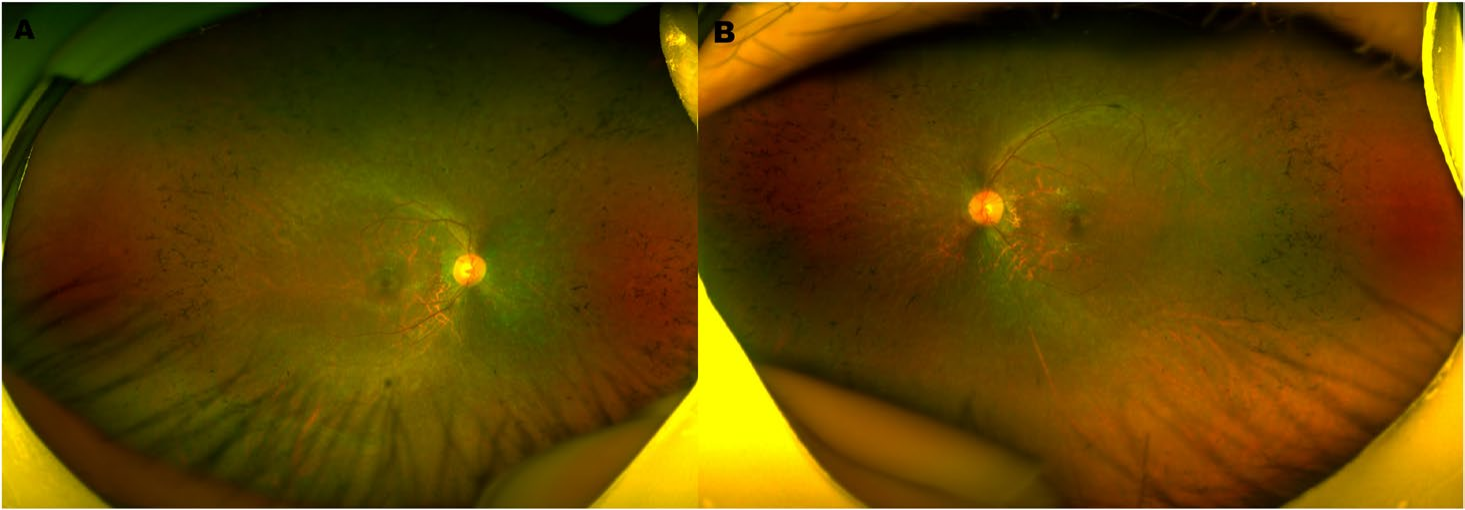
Fundus photographs. Bilateral optic discs show waxy pallor with attenuated vessels and extensive bone‐spicule pigmentary deposits in the peripheral retina.

Fundus examination (Figure 1) demonstrated bilateral optic discs with a waxy pallor, attenuated retinal vessels, and extensive bone‐spicule pigmentary deposits distributed across the peripheral retina, consistent with classic RP.

**FIGURE 2:**
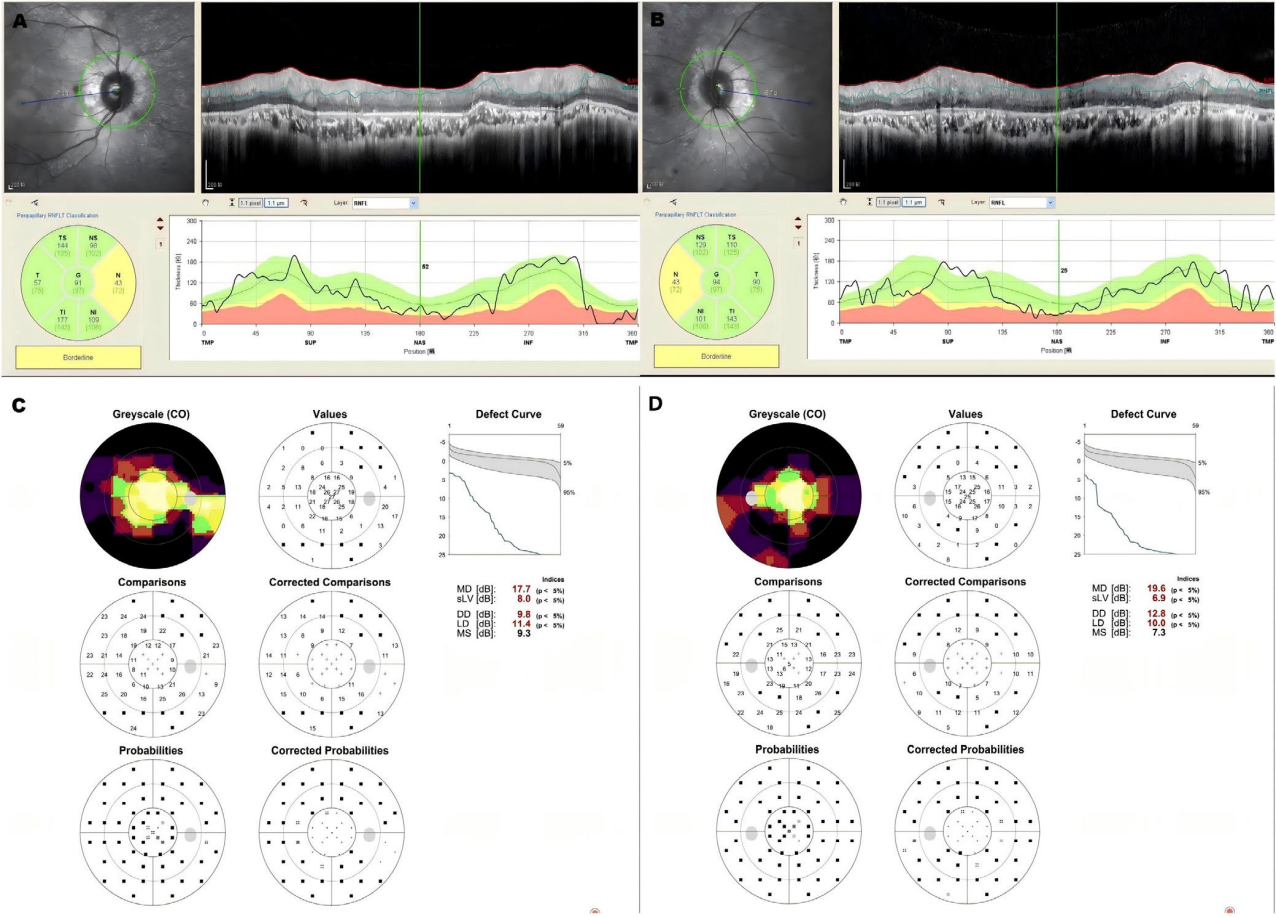
OCT and Visual Field. (A, B) OCT imaging demonstrates thinning of the nasal retinal nerve fiber layer. (C, D) Visual field testing reveals severe bilateral concentric constriction (tubular vision).

Optical Coherence Tomography (OCT) of the peripapillary retinal nerve fiber layer (RNFL) (Figure 2A,B) demonstrated isolated nasal quadrant thinning bilaterally (43 μm; normal range: 85–120 μm). Automated perimetry (Figure 2C,D) revealed bilateral concentric constriction of the visual field (tubular vision).

### 2.3 Genetic Testing

**FIGURE 3:**
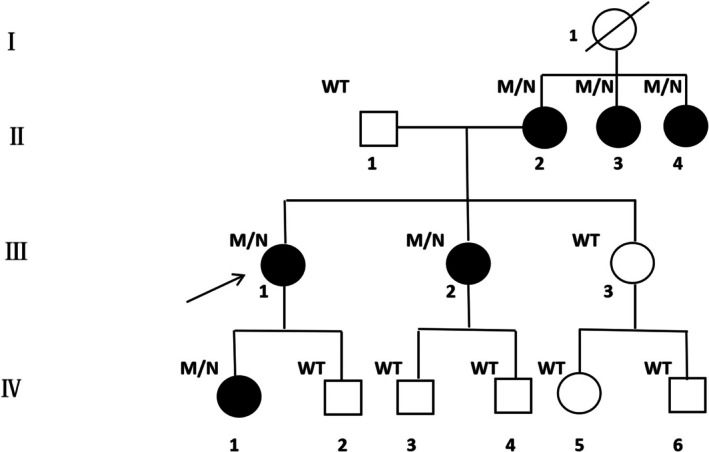
Family Pedigree. The pedigree shows the autosomal dominant inheritance pattern. Filled symbols indicate affected individuals with the PRPF6 c.514C>T variant.

Genomic DNA was extracted from peripheral blood. Whole‐Exome Sequencing (WES) identified a heterozygous c.514C>T variant in the *PRPF6* gene in the proband (Figure 3). This variant results in an amino acid change from Arginine to Tryptophan (p.Arg172Trp).

**FIGURE 4:**
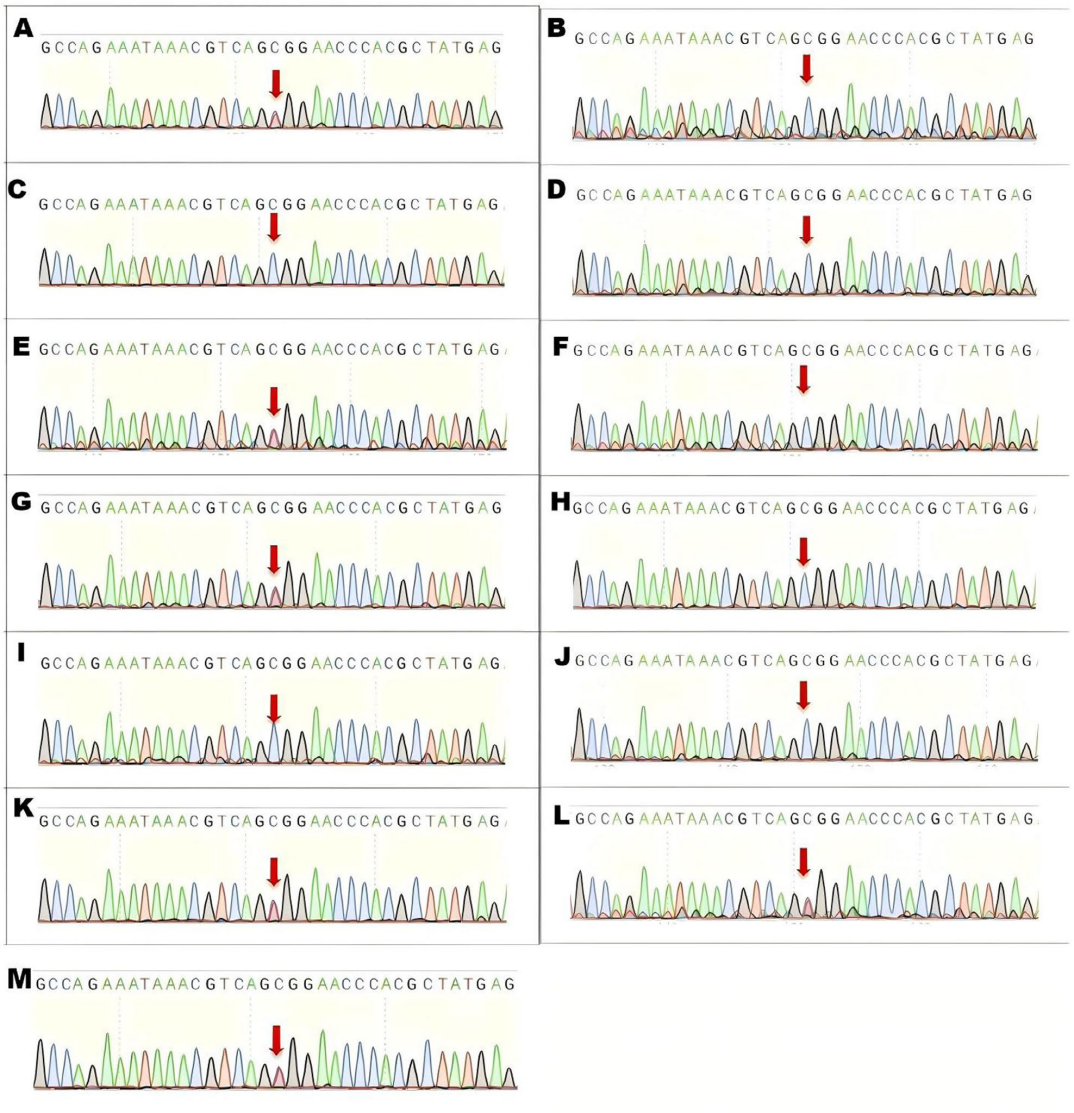
Sanger Sequencing. Chromatograms showing the PRPF6 gene region. (A) The proband showing the heterozygous c.514C>T mutation (arrow), resulting in p.Arg172Trp. (E) The proband's mother showing the same heterozygous variant. (L, M, G) Other affected family members carrying the variant. (B, C, D, F, H, I, K) Non‐carriers showed homozygous wild‐type genotypes.

Sanger sequencing (Figure 4) confirmed the presence of the variant. Segregation analysis revealed that the variant was inherited from the proband's mother (affected). Among the siblings, the affected sister carried the variant, while unaffected siblings did not. The variant co‐segregated perfectly with the disease phenotype across the tested family members.

## 3 Conclusion and Results

Based on clinical and genetic findings, the variant was classified as Likely Pathogenic according to ACMG guidelines.

## 4 Discussion

This case documents the discovery of the *PRPF6* c.514C>T (p.Arg172Trp) variant in an adRP family. The *PRPF6* protein N‐terminal TPR domain is responsible for spliceosome subunit recognition, while the C‐terminal HEAT repeat motifs maintain U5 snRNP stability [5].

Previously reported pathogenic variants have predominantly been located in the C‐terminal HEAT repeat region [4, 6]. The identification of this novel N‐terminal variant suggests that disruption of the TPR domain can also lead to clinical disease, likely by affecting spliceosome subunit recognition and assembly rather than stability alone.

Clinically, typical C‐terminal mutations (e.g., c.2699G>A) are often associated with early macular atrophy [7, 8]. In contrast, this patient retained 20/20 central vision at age 41, with structural preservation of the central retina despite severe peripheral field loss. This suggests a potential genotype–phenotype correlation where N‐terminal variants might exhibit a “macula‐sparing” phenotype compared to their C‐terminal counterparts, though further functional studies are required to validate this hypothesis.

Currently, many RP gene testing panels focus on hotspot regions and may miss the N‐terminal region of *PRPF6*. This study suggests the necessity of expanding testing ranges to include the full coding region of *PRPF6*.

## Author Contributions

**Na Li:** data curation, formal analysis, software, writing – original draft. **Yalong Dang:** funding acquisition, investigation, methodology, project administration, resources, supervision, visualization, writing – review and editing.

## Funding

This study was supported by The Key Project of Sanmenxia City (2022001007, recipient: Yalong Dang) and The Education and Research Project of Henan Province (Wjlx2022165, recipient: Yalong Dang).

## Conflicts of Interest

The authors declare no conflicts of interest.

## Data Availability

The data supporting this study's findings are not publicly available for privacy reasons but from the corresponding author upon reasonable request.
